# Changes in fat uptake, color, texture, and sensory properties of *Aloe vera* gel‐coated eggplant rings during deep‐fat frying process

**DOI:** 10.1002/fsn3.3238

**Published:** 2023-01-27

**Authors:** Mehdi Varidi, Saba Ahmadzadeh‐Hashemi, Majid Nooshkam

**Affiliations:** ^1^ Department of Food Science and Technology Faculty of Agriculture Ferdowsi University of Mashhad (FUM) Mashhad Iran

**Keywords:** *Aloe vera*, edible coating, eggplant, oil uptake, sensory properties

## Abstract

There is a widespread use of deep‐fat frying in both domestic and industrial sections, and deep‐fat fried foods are extremely popular due to their taste, color, and crispy texture. Human health can be, however, seriously compromised by the excessive consumption of oil, especially saturated fats and trans fatty acids. The use of hydrocolloids in inhibiting oil absorption has garnered considerable attention. This study was therefore aimed to lower the oil absorption in eggplant rings during the deep‐fat frying process with the aid of *Aloe vera* gel coating. The effects of gel concentration (0%, 50%, and 100%), frying time (2, 5, and 8 min), and frying temperature (160°C and 180°C) on the oil uptake, moisture content, texture, color, and sensory properties of the eggplant rings were evaluated. The gel coating led to a decrease in oil uptake (up to 50%), hardness (up to 0.98‐fold), Δ*E* (up to 0.89‐fold), and overall acceptance (up to 0.85‐fold), and an increase in moisture content (up to 1.47‐fold) and lightness (up to 1.14‐fold) of the samples. The frying time and temperature also influenced the physiochemical and sensory properties of the eggplant rings. The sample coated with 50% gel and fried at 180°C for 8 min had lower oil content and water loss with the highest acceptance rate in terms of taste, color, odor, texture, and appearance. The *Aloe vera* gel could be, therefore, a good candidate with high nutritional and economic value to reduce oil uptake in fried food products.

## Graphical abstract

The sample coated with 50% gel and fried at 180°C for 8 min had lower oil content and water loss with the highest acceptance rate in terms of taste, color, odor, texture, and appearance. The *Aloe vera* gel could be a good candidate with high nutritional and economic value to reduce oil uptake in fried food products.

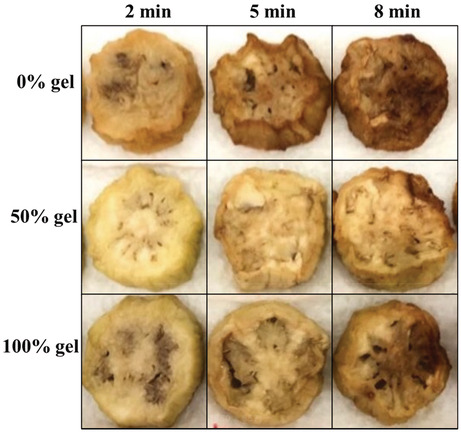

## 1 INTRODUCTION

Deep‐fat frying is one of the oldest and fastest methods of food preparation that is widely used on both domestic and industrial scales. The taste, color, and crispy texture of deep‐fat fried foods make them very popular among consumers (Boskou et al., 2006). Frying is a process of immersing food in hot oil with contact among oil, air, and food at high temperatures ranging from 150°C to 190°C. A fried food's desirable and unique quality is produced by the simultaneous heat and mass transfer of oil, food, and air during deep‐fat frying. The frying oil acts as a heat transfer medium and provides flavor and texture to fried foods (Asokapandian et al., 2020; Choe & Min, 2007).

The amount of fat absorbed during the frying process is one of the most important quality parameters for fried foods, which determines the trend toward healthier food and low‐fat products (Bouchon, 2009). The excessive consumption of oil, especially saturated fats and trans fatty acids, is a major threat to human health and can cause diseases such as high blood pressure, high cholesterol, coronary artery blockages, and obesity (DiNicolantonio et al., 2016; Siri‐Tarino et al., 2010). Low‐fat products are therefore preferred by consumers.

The reduction of oil in deep‐fat‐fried products can be achieved by pre‐frying and/or post‐frying treatments. Pre‐frying treatments aim to reduce surface permeability due to the marked effect that crust microstructure has on oil absorption. However, the purpose of post‐frying treatments is to remove surface oil before the suction of post‐cooling begins (Bouchon, 2009). Hydrocolloids have, however, attracted considerable attention over the last decade for their use in inhibiting oil absorption (Kurek et al., 2017; Salehi, 2020).

Hydrocolloids form a uniform coating over the surface of foods during deep fat frying, which prevents excessive oil absorption. They provide limited moisture permeability, function as oxygen and carbon dioxide barriers, and protect against lipid oxidation due to their high hydrophilicity (Alizadeh Behbahani et al., 2021; Asokapandian et al., 2020; Heydari et al., 2020; Noshad et al., 2021; Tanavar et al., 2021). Mechanical and barrier properties of a coating, along with characteristics of the food substrate, determine its effectiveness (García et al., 2002). The effect of xanthan (Asadnahal et al., 2022), sage seed gum (Salehi & Asadnahal, 2022), and alginate (Bagheri et al., 2022) on the oil absorption of food products during frying has been studied.

The herbal plant *Aloe vera* Linné (also known as *Aloe barbadensis* Miller) has been extensively studied for its potential use in food, pharmaceuticals, and cosmetics (Maan et al., 2018). The *Aloe vera* gel is composed of soft and slippery tissues containing parenchyma cells. This jelly‐like material is transparent and contains many bioactive compounds, including carbohydrates (mannans, galactans, pectic substances, xylan, cellulose), proteins, amino acids, soluble sugars, fibers, minerals, vitamins, organic acids, and polyphenols (Sharrif Moghaddasi & Verma, 2011). It is used in the food industry for the preparation of functional foods, as a natural preservative, or as a material for edible films and coatings (Maan et al., 2018). Due to its effectiveness in extending the shelf‐life of various perishable food commodities, it has received considerable attention over the last decade as an edible film/coating. *Aloe vera* gel films/coatings are an excellent example of natural and active packaging not only because of their barrier properties but also because of their antioxidative and antimicrobial properties (Maan et al., 2021). It can also be used as a barrier to prevent oil absorption during deep‐fat frying. The application of nanoemulsions of eugenol oil with *Aloe vera* gel on shrimps is reported by Sharifimehr et al. (2019). Deep‐fat frying of shrimp decreased oil absorption and cooking loss. The nanoemulsion coating significantly reduced drip loss during cold storage and improved antioxidative activity (Sharifimehr et al., 2019). Besides, *Aloe vera* gel coating is sufficient for delaying browning and protecting the overall quality of fruits during storage (Ali et al., 2019).

Eggplant (*Solanum melongena* L.) belongs to the family *Solanaceae* and the genus *Solanum*. It is highly low in calories (11 calories/100 g), so it is very useful in a slimming diet. Also, it is an excellent source of antioxidants and phenolic compounds (Uthumporn et al., 2016). The fruit is commonly used in cooking and frying and is highly capable of absorbing large amounts of frying oils (Salehi, 2020; Salehi & Asadnahal, 2022). Pre‐treatments and gum‐based edible coatings have been used to reduce oil absorption during eggplant frying (Khaliliyan et al., 2017; Salehi & Asadnahal, 2022). However, to the best of our knowledge, there is no research in the literature regarding the use of *Aloe vera* gel coating to lower oil absorption of eggplant slices during the frying process. Therefore, this study aimed to evaluate the effect of *Aloe vera* gel‐based edible coating on the fat uptake, color, texture, and sensory properties of eggplant rings during the deep‐fat frying process.

## 2 MATERIALS AND METHODS

### 2.1 Materials

Eggplants were purchased from a local market. They were washed, peeled, and kept at 4°C until the tests were performed.

### 2.2 Preparation of coating solutions

In order to prepare colloidal suspensions, the *Aloe vera* plant was purchased from a local market and after peeling, the gel or its central part was separated and mixed by a household mixer. The coating solutions were 0% gel (non‐coated), 50% gel (diluted gel; gel‐to‐water ratio of 1:1), and 100% gel (non‐diluted gel).

In order to measure the viscosity and flow behavior constants of the coating solutions, a Brookfield viscometer (LVDV III‐ULTRA, Brookfield) was used. LV2 spindle at 5–250 rpm was used for the measurements. The flow behavior constants of the samples were determined by fitting the shear stress and rate data to the Power‐law model (Lavaei et al., 2022; Nooshkam et al., 2023):

$$\tau =K\gamma^{n}$$

where *K* is the consistency index (Pa.s^n^) and *n* is the flow behavior index (dimensionless).

### 2.3 Preparation of eggplant rings

Eggplants were cut into pieces with a diameter of 2.5 cm and a thickness of 1.0 cm. The samples were immersed in the coating solutions for 1.0 min and then placed on a strainer to remove the excess solution (Sari et al., 2017).

### 2.4 Frying process

The coated and uncoated samples were fried in a DeLonghi deep fryer (F38436) with frying sunflower oil (Ladan) at 160°C and 180°C for 2, 5, and 8 min. After frying, the samples were placed on a strainer to remove excess oil.

### 2.5 Fried eggplant characterization

#### 2.5.1 Moisture content

The moisture content of the fried samples was measured according to the AOAC standard method by placing the samples in an oven at a temperature of 105°C until reaching a constant weight (AOAC, 2000).

#### 2.5.2 Oil content

The amount of oil absorption of the fried eggplants was determined by the Soxhlet method (Sari et al., 2017).

#### 2.5.3 Texture

A cylindrical probe was used to compress the samples at a test speed of 1.0 mm s^−1^, by a texture analyzer (Stable Microsystems, TA‐XT plus). The hardness of the samples was measured from force‐time curves.

#### 2.5.4 Color

The color indices (L*, a*, and b*) of the fried eggplants were measured by a chromameter (Konica Minolta, C‐410). A standard tile was used to calibrate the device, and the color indices were then recorded to calculate the total color difference (Δ*E*), whiteness index (WI), and °H of the samples, as below (Nooshkam et al., 2022):

(1) $$∆E=\sqrt{∆L{*}^{2}+∆b{*}^{2}+∆a{*}^{2}}$$

(2) $$\mathrm{WI}=100-{\left[\left(100-L{*}^{2}\right)+\left(a{*}^{2}+b{*}^{2}\right)\right]}^{1/2}$$

(3) $$H{}^{\circ}=\tan^{-1}\left(\frac{b^{*}}{a^{*}}\right)$$

#### 2.5.5 Sensory evaluation

The sensory properties (appearance, color, odor, texture, taste, and overall acceptance) of fried eggplant samples were evaluated by a 12‐member panel using the 5‐point hedonic scale test (1, extremely dislike; 2, slightly dislike; 3, neither like nor dislike; 4, slightly like; 5, extremely like) (Jouki & Khazaei, 2022).

### 2.6 Statistical analysis

The data were analyzed by Minitab software (version 19) using a completely randomized design in a factorial arrangement. The factors were gel concentration (0%, 50%, and 100%), frying temperature (160°C and 180°C), and frying time (2, 5, and 8 min). The significant differences between the means were evaluated by the Tukey test at *p* < .05. The experiments were performed in three replicates.

## 3 RESULTS AND DISCUSSION

### 3.1 Rheological properties of coating solutions

**FIGURE 1:**
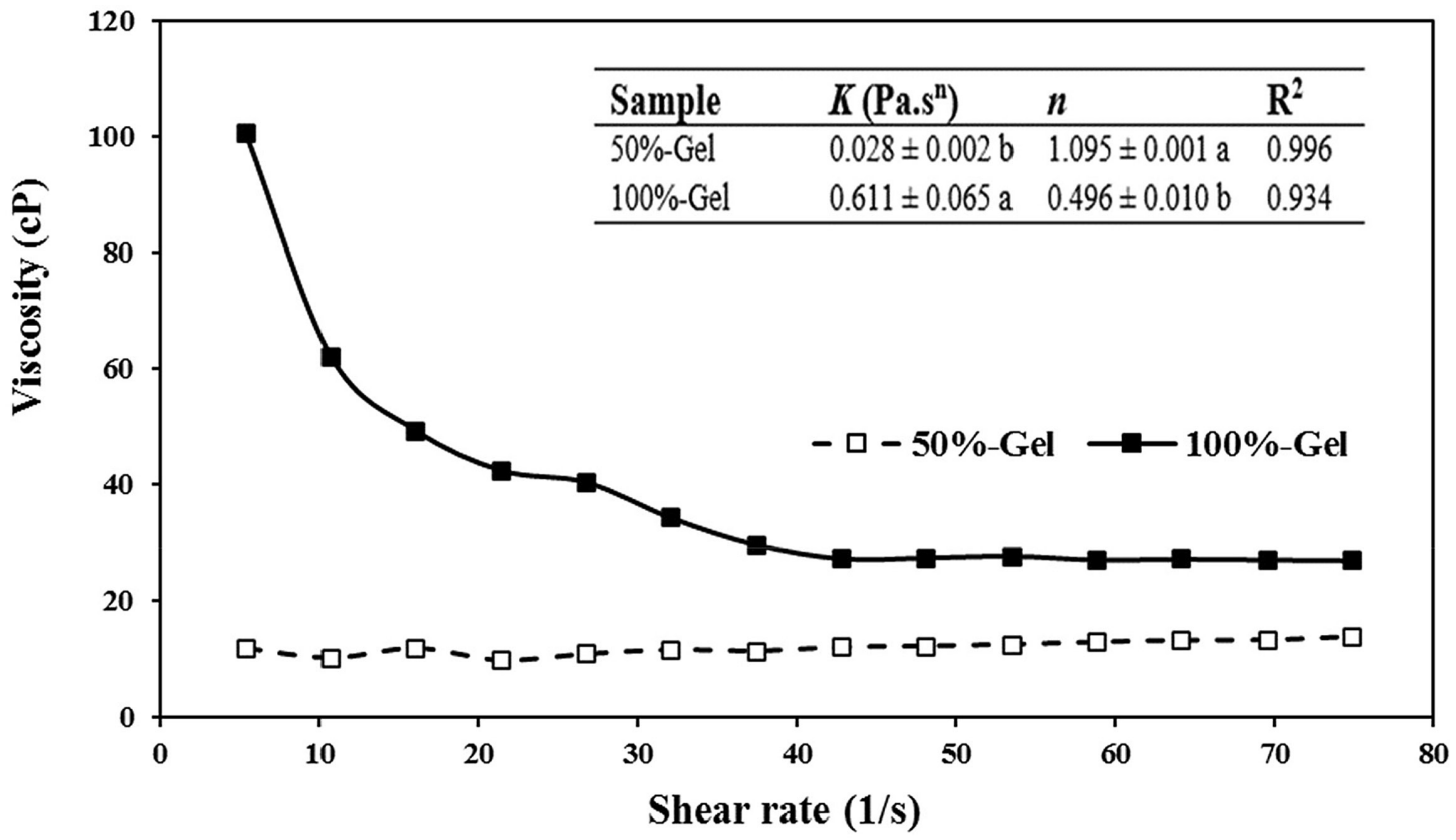
Rheological properties of coating solutions. Different letters indicate significant differences between samples at *p* < .05.

Figure 1 shows the changes in apparent viscosity of coating solutions as a function of shear rate. The apparent viscosity of *Aloe vera* gel (i.e., 100%‐gel) decreased markedly as the shear rate increased, indicating a shear thinning behavior (*K* ~ 0.61 and *n* ~ 0.50). However, diluting the gel resulted in a polysaccharidic solution (i.e., 50%‐gel) with a Newtonian behavior (*K* ~ 0.03 and *n* ~ 1.0); the apparent viscosity of the solution did not change with shear rate. Generally, lower water content causes a higher viscosity (Xue & Ngadi, 2006), and the non‐Newtonian behavior of 100%‐gel might be due to its higher glucomannan content (Suriati et al., 2020).

### 3.2 Moisture content

**FIGURE 2:**
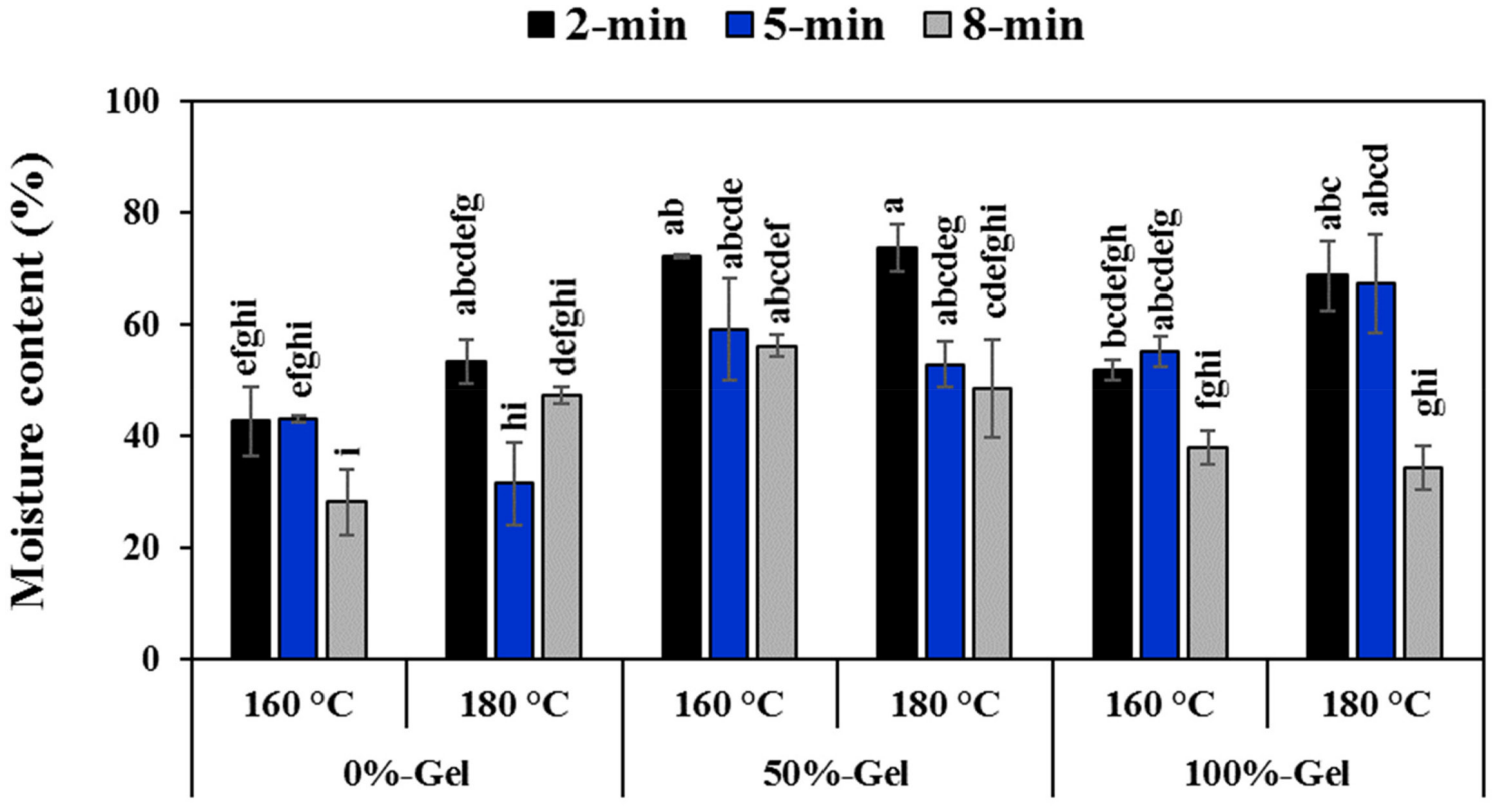
Changes in moisture content of fried eggplant rings as a function of gel concentration, frying temperature, and frying time. Different letters indicate significant differences between samples at *p* < .05.

The moisture content of the fried eggplants was significantly influenced by gel concentration (Figure 2). It rose remarkably from 41.06% to 60.52% as the gel concentration changed from 0% to 50% (*p* < .05), but further increase in gel concentration (i.e., 100%) led to a lower moisture content (52.67%) (*p <* .05). The poor performance of 100% gel coating can be due to the fact that during frying and when moisture is removed from the samples, the amount of water in the coating increases, and because water can act as a plasticizer, it reduces the hydrogen bonds between the polymer chains of the coating. Then intermolecular spaces could be increased and the barrier property of the coating against water can be decreased (Bagheri et al., 2019; Berenji et al., 2011). Likewise, it has been reported that coating eggplant rings with higher concentrations of guar led to lower moisture content in the fried sample (Jorjani & Hamrahi, 2015). Prober concentration of *Aloe vera* gel could act as a barrier to maintain moisture and prevent water evaporation during the frying process (Sumnu & Sahin, 2008). Similarly, fried chicken meat coated with *Aloe vera* gel illustrated moisture retention compared to the control sample (Sari et al., 2017). This may correlate to the high apparent viscosity of the coat or its high adherence (Salehi, 2020).

The frying time also had a significant effect on the moisture content of the samples (Figure 2). Moisture content decreased from 60.49% to 42.15% as the frying time raised from 2 to 8 min (*p* < .05). Moisture content during frying generally decreases exponentially with frying time (Sumnu & Sahin, 2008). In congruent with our results, Varghese et al. (2014) reported that longer frying increased water loss remarkably. The reason behind the water loss by the frying time is probably accorded to the formation of a dehydrated zone, a brittle layer in which the evaporation of free water and bound water is occurring by heating (Eissa et al., 2013).

The frying temperature did not noticeably influence the moisture content (*p* > .05); however, samples fried at 180°C had higher moisture content than those obtained at 160°C (53.14% vs. 49.71%). This could be due to the fact that by increasing the frying temperature, the surface of the samples dries up and forms a crust (case hardening), preventing moisture loss (Krokida et al., 2000b). It has similarly been found that temperature has no marked effect between 150°C and 180°C (Sumnu & Sahin, 2008). Generally, the highest and lowest moisture contents (73.72% vs. 28.15%) were observed in 50‐2‐180 and 0‐8‐160 samples, respectively.

### 3.3 Oil content

**FIGURE 3:**
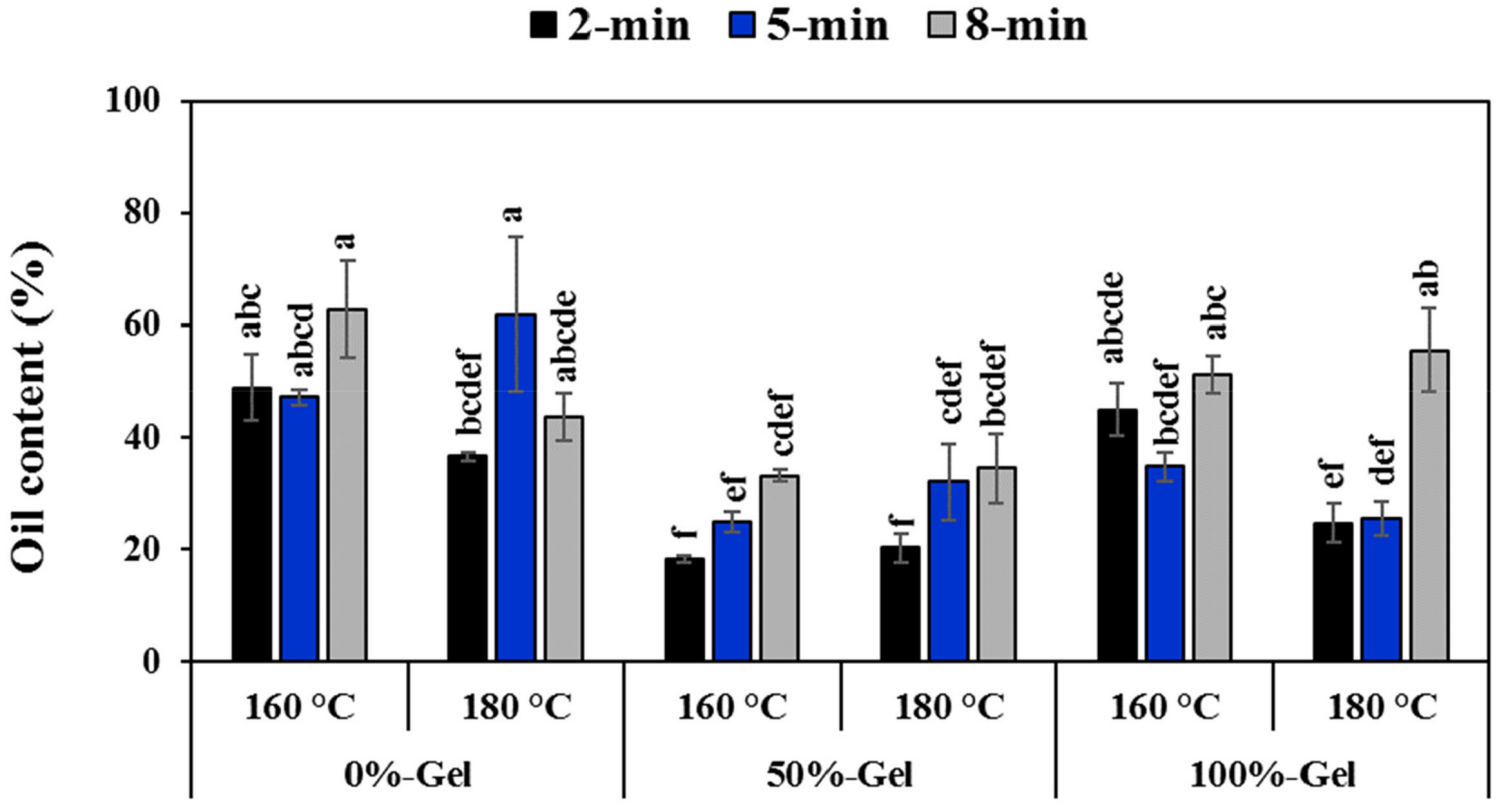
Changes in oil content of fried eggplant rings as a function of gel concentration, frying temperature, and frying time. Different letters indicate significant differences between samples at *p* < .05.

Figure 3 illustrates the oil content of the coated and uncoated fried eggplants. By increasing *Aloe vera* gel concentration from 0% to 50%, oil content decreased by an average of 50% (*p* < .05). However, a further increase in gel concentration (100% gel) resulted in higher oil content, but it was still significantly lower than the control sample. This could be likely due to the barrier function of *Aloe vera* gel which leads to less oil absorption (Sumnu & Sahin, 2008). By the same token, Archana et al. (2016) observed a marked reduction in oil uptake in the fried sample by a mixture of Okra and carrageenan gums.

By increasing the frying time from 2 to 8 min, the amount of oil went up significantly from 32.31% to 46.85% (*p* < .05). Similarly, it has been revealed that oil content went up with frying time in both treated and untreated samples until it reaches equilibrium (Krokida et al., 2000a). It is also worth mentioning that the oil content can increase during cooling time (Sumnu & Sahin, 2008), and capillary forces, rather than vapor condensation, are responsible for oil uptake during the cooling period (Ziaiifar, 2008).

Oil content is directly dependent on the temperature of the frying process (Sumnu & Sahin, 2008). Although the main effect of temperature on the oil content was not significant (*p* > .05), the oil content of the samples decreased with increasing temperature (40%–38%), which may be due to the case hardening effect (Krokida et al., 2000b), as surface wetting is a crucial mechanism of oil absorption (Moyano & Pedreschi, 2006). In general, this behavior agrees with the hypothesis that higher frying temperatures lead to lower oil absorption (Sumnu & Sahin, 2008). This may be due to the quick pressure build‐up during frying at high temperatures, which prevents capillary diffusion of oil (Yamsaengsung & Moreira, 2002).

According to the results, the coated fried eggplants with a gel concentration of 50% which were fried at 160°C for 2 min (50‐2‐160) contained the least oil content (18.30%). The uncoated fried sample at a lower temperature for the longest time gained the highest oil uptake (62.90%). It could be also noteworthy that samples with less oil content contained more moisture content.

### 3.4 Hardness

Due to the importance of texture on the consumer's perception of foods, the hardness of the coated and uncoated fried eggplant rings was measured, and the results are provided in Table 1. The hardness of the coated and uncoated samples was little influenced by gel concentration. All samples' hardness was about 3200 *g* (*p* > .05). However, the slight decrease in the hardness of the samples (up to 0.98‐fold), as a function of gel concentration, may be related to the ability of coating in preserving the moisture content of the product during the frying process (Salehi, 2020).

**TABLE 1:** The effect of coating with *Aloe vera* gel on the hardness of fried eggplant rings.

Gel concentration (%)	Time (min)	Temperature (°C)	Hardness (*g*)
0	2	160	1501 ± 142^ef^
		180	5655 ± 489^a^
	5	160	3230 ± 347^bcdf^
		180	2607 ± 640^cdef^
	8	160	1495 ± 172^ef^
		180	5169 ± 154^ab^
50	2	160	2273.8 ± 78^def^
		180	4765 ± 1267^abc^
	5	160	3026 ± 626^bcdef^
		180	5067 ± 271^ab^
	8	160	2102 ± 210^def^
		180	2344 ± 336^def^
100	2	160	4075 ± 1119^abcd^
		180	4253 ± 880^abcd^
	5	160	3652 ± 581^abcde^
		180	4295 ± 691^abcd^
	8	160	1640 ± 181^ef^
		180	1304 ± 352^f^

*Note*: Means within the same column with different superscripts differ significantly (*p* < .05).

The hardness of all samples reduced significantly from 3753 *g* to 2342 *g* as the frying time rose from 2 to 8 min (*p* < .05). Similarly, the samples fried at 180°C had significantly greater hardness values than those obtained at 160°C (3940 *g* vs. 2554.74 *g*). This could be due to the case‐hardening effect that occurred at higher frying temperatures/times (Salehi & Asadnahal, 2022). The highest (5654.92 *g*) and lowest (1303.93 *g*) hardness values accounted for 0‐2‐180 and 100‐8‐180 samples, respectively. It can generally be seen that the coated samples with higher moisture content had lower hardness values, as well.

### 3.5 Color

Color is one of the crucial parameters in food products, which reflects their sensory and quality properties. The brightness and WI of the uncoated sample were lower than other samples (Table 2). Among the coated ones, the sample with higher gel concentration had a brighter color (*p* < .05), which presented the optimal concentration of *Aloe vera* gel to protect samples against color changes (Table 2). This could be attributed to the higher moisture content of the gel‐coated samples (Kenari et al., 2019). In contrast, as the frying time and temperature increased, the fried samples became darker and Δ*E* went up (*p* < .05). Similarly, by increasing the frying time, the L* index decreased, which could be due to the increase in the browning reaction rate (Bagheri et al., 2022). Also, Ali et al. (2011) announced the highest lightness and the least color changes for eggplant slices coated with 2% chitosan (Ali et al., 2011).

**TABLE 2:** The effect of coating with *Aloe vera* gel on the color parameters of fried eggplant samples.

Gel concentration (%)	Time (min)	Temperature (°C)	L*	a*	b*	Δ*E*	WI	Hue angle (H°)
0	2	160	69.15 ± 0.22^ab^	2.03 ± 0.82^abc^	20.71 ± 0.44^a^	37.52 ± 0.03^b^	62.78 ± 0.02^a^	1.47 ± 0.04^b^
		180	58.46 ± 8.56^bcdf^	6.72 ± 5.02^abc^	22.18 ± 5.15^a^	48.3 ± 5.69^ab^	51.95 ± 5.72^ab^	1.26 ± 0.27^bcde^
	5	160	60.74 ± 2.37^bcd^	5.90 ± 1.46^abc^	19.76 ± 1.33^a^	44.65 ± 1.68^ab^	55.63 ± 1.70^ab^	1.28 ± 0.03^bcde^
		180	49.61 ± 6.20^def^	9.14 ± 0.97^a^	14.28 ± 5.58^a^	53.72 ± 4.54^ab^	46.53 ± 4.52^ab^	0.96 ± 0.23^def^
	8	160	54.07 ± 0.46^cdef^	8.26 ± 1.87^ab^	15.05 ± 6.28^a^	49.48 ± 2.62^ab^	50.79 ± 2.66^ab^	1.05 ± 0.09^cdef^
		180	45.59 ± 0.25^f^	8.87 ± 1.77^ab^	7.23 ± 0.23^a^	55.91 ± 0.47^a^	44.38 ± 0.495^b^	0.69 ± 0.11^f^
50	2	160	74.12 ± 1.76^a^	−1.02 ± 0.00^bc^	27.08 ± 7.17^a^	38.55 ± 3.97^ab^	61.76 ± 4.02^ab^	179.96 ± 0.01^a^
		180	67.20 ± 2.70^abc^	1.58 ± 0.57^abc^	27.2 ± 8.29^a^	43.14 ± 7.34^ab^	57.15 ± 7.38^ab^	1.51 ± 0.00^b^
	5	160	69.29 ± 0.060^ab^	3.37 ± 0.68^abc^	25.55 ± 8.87^a^	40.64 ± 5.57^ab^	56.62 ± 5.62^ab^	1.44 ± 0.02^bc^
		180	54.92 ± 2.96^cdef^	9.30 ± 4.48^a^	19.01 ± 8.50^a^	50.32 ± 6.65^ab^	49.92 ± 6.73^ab^	1.12 ± 0.02^bcde^
	8	160	60.51 ± 3.02^bcde^	7.51 ± 3.77^abc^	23.29 ± 11.63^a^	47.12 ± 8.85^ab^	53.12 ± 8.93^ab^	1.26 ± 0.00^bcde^
		180	47.35 ± 5.07^ef^	10.28 ± 4.62^a^	12.59 ± 3.60^a^	55.44 ± 6.46^a^	44.82 ± 6.52^b^	0.90 ± 0.09^ef^
100	2	160	76.16 ± 3.44^a^	−2.22 ± 1.34^c^	27.09 ± 4.75^a^	36.71 ± 1.12^b^	63.61 ± 1.20^a^	179.91 ± 0.06^a^
		180	70.69 ± 1.14^ab^	2.84 ± 1.33^abc^	26.20 ± 2.43^a^	39.71 ± 0.81^ab^	60.54 ± 0.86^ab^	1.47 ± 0.04^b^
	5	160	65.69 ± 0.21^abc^	4.29 ± 1.46^abc^	17.96 ± 2.99^a^	39.30 ± 1.68^ab^	60.99 ± 1.72^ab^	1.34 ± 0.40^bcd^
		180	59.82 ± 3.01^bcde^	8.26 ± 2.11^ab^	21.26 ± 0.73^a^	46.47 ± 2.63^ab^	53.76 ± 2.65^ab^	1.20 ± 0.97^bcde^
	8	160	60.67 ± 1.08^bcd^	7.32 ± 2.15^abc^	19.81 ± 5.53^a^	45.07 ± 1.79^ab^	55.17 ± 1.85^ab^	1.22 ± 0.00^bcde^
		180	51.83 ± 0.092^def^	9.05 ± 2.74^ab^	12.12 ± 4.24^a^	50.87 ± 1.37^ab^	49.39 ± 1.42^ab^	0.93 ± 0.02^ef^

*Note*: Means within the same column with different superscripts differ significantly (*p* < .05).

**FIGURE 4:**
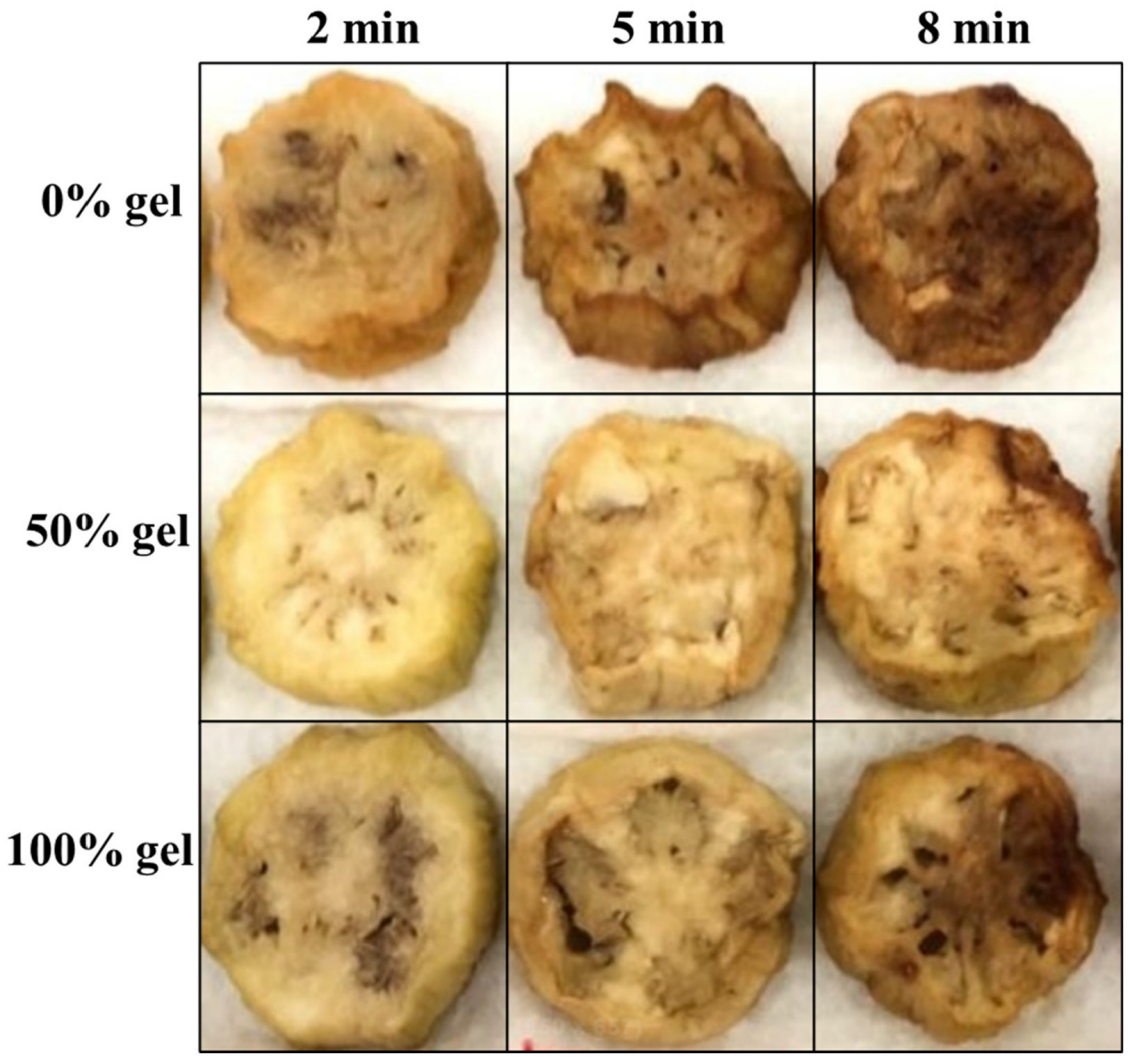
Visual observation of uncoated and coated eggplant rings fried at 160°C.

The uncoated samples had significantly higher H° (yellower) compared to the coated eggplant (*p* < .05), as greatly confirmed by the visual observation (Figure 4). Although, a* index did not change significantly with the increase in gel concentration (*p* > .05), the gel‐coated samples had lower redness index compared to the uncoated counterparts. The frying time and temperature had a different effect on the color indices of the eggplants, and samples fried at 180°C for 8 min were clearly redder than those fried at 160°C in shorter times (*p* < .05). The yellowness index (b*) of the samples declined as a function of frying time and temperature. It could be noteworthy that different coatings have different results on the redness and yellowness of samples (Salehi, 2020). For example, the application of protein‐based coatings increased the yellowness of eggplant (Bagheri et al., 2022); however, xanthan‐based coating resulted in a decrease in the yellowness of the eggplant during the deep‐fat frying process (Asadnahal et al., 2022). The coated samples had generally lower color changes in comparison to the non‐coated ones, which is in good agreement with the sensory acceptance by the panelists (Table 3).

**TABLE 3:** The effect of coating with *Aloe vera* gel on the sensory properties of fried eggplant rings.

Gel concentration (%)	Time (min)	Temperature (°C)	Appearance	Color	Odor	Taste	Texture	Overall acceptability
0	2	160	3.00 ± 0.43^bc^	3.00 ± 0.60^cde^	4.50 ± 0.67^ab^	3.00 ± 0.95^bc^	2.17 ± 0.58^def^	3.08 ± 0.52^c^
		180	2.50 ± 1.00^cd^	2.67 ± 0.89^def^	3.67 ± 1.23^ab^	3.17 ± 0.94^bc^	2.75 ± 0.87^cde^	2.83 ± 0.84^cd^
	8	160	4.50 ± 0.52^a^	4.25 ± 0.45^ab^	4.58 ± 0.52^ab^	4.08 ± 0.52^ab^	3.92 ± 0.79^ab^	4.08 ± 0.52^ab^
		180	4.58 ± 0.52^a^	4.33 ± 0.49^ab^	4.67 ± 0.65^ab^	4.75 ± 0.45^a^	4.58 ± 0.52^a^	4.67 ± 0.49^a^
50	2	160	1.92 ± 0.99^d^	1.92 ± 0.90^f^	3.92 ± 1.24^ab^	2.08 ± 1.16^c^	1.67 ± 0.49^f^	1.92 ± 0.67^d^
		180	2.50 ± 1.31^cd^	2.17 ± 1.03^ef^	3.42 ± 1.68^b^	3.25 ± 0.62^bc^	2.83 ± 0.83^cde^	3.00 ± 0.95^c^
	8	160	3.42 ± 0.79^bc^	3.42 ± 0.79^bcd^	3.83 ± 1.19^ab^	3.08 ± 1.08^bc^	3.00 ± 0.85^bcd^	3.17 ± 0.84^bc^
		180	4.92 ± 0.29^a^	5.00 ± 0.00^a^	5.00 ± 0.00^a^	4.92 ± 0.29^a^	4.75 ± 0.45^a^	5.00 ± 0.00^a^
100	2	160	2.58 ± 0.79^cd^	2.67 ± 0.65^def^	3.92 ± 1.17^ab^	2.58 ± 1.08^c^	2.08 ± 0.52^def^	2.75 ± 0.62^cd^
		180	2.42 ± 0.52^cd^	2.17 ± 0.84^ef^	3.42 ± 1.67^b^	2.58 ± 0.99^c^	2.00 ± 0.60^ef^	2.58 ± 0.52^cd^
	8	160	2.58 ± 0. 99^cd^	2.75 ± 0.75^def^	3.58 ± 1.56^ab^	3.00 ± 0.60^bc^	3.33 ± 0.49^bc^	3.17 ± 0.72^bc^
		180	3.92 ± 0.67^ab^	3.92 ± 0.52^bc^	4.25 ± 1.06^ab^	3.83 ± 1.03^ab^	3.83 ± 0.94^ab^	4.08 ± 0.90^ab^

*Note*: Means within the same column with different superscripts differ significantly (*p* < .05).

### 3.6 Sensory evaluation

The results of the sensory evaluation of fried eggplants are provided in Table 3. The changes in appearance, color, odor, taste, and texture of the samples were in the same line; samples coating with higher gel concentration (from 0% to 100%) declined acceptance in each item, remarkably (*p* < .05). On the other hand, increasing the time and temperature of frying showed a great upward trend in the willingness of the panelists to eat eggplants (*p* < .05). It is worth mentioning that, at low frying temperatures, the cooking time is lengthened to obtain the desired color in the food, leading to an increase in oil uptake (Melton, 2007). In the present research, less rigid and redder samples were more popular which were in line with texture and colorimetry findings. Surprisingly, in all the samples, the changes in frying temperature in terms of odor did not experience any significant difference from each other and they almost got the same score in the sensory evaluation (*p* > .05); however, longer frying led to a desirable odor in eggplants (*p* < .05). Sensory responses indicated that 50%‐gel coated eggplant fried at 180°C for 8 min received the highest sensory scores, while by reducing the time and temperature of frying to 2 min and 160°C with 50% gel concentration, the most undesirable sample induced (*p* < .05).

## 4 CONCLUSION

This study was conducted to study water and oil content besides hardness and sensory evaluation to select the best frying conditions in coated and uncoated eggplant samples with proper frying time and temperature. It was demonstrated that by applying the appropriate conditions of the frying process, frying time of 8 min, *Aloe vera* gel concentration of 50%, and frying temperature of 180°C, a product with lower oil content and water loss with the highest acceptance rate in terms of taste, color, odor, texture, and appearance was gained. Moreover, the current improved features make the *Aloe vera* gel a good candidate with high nutritional and economic value to reduce oil uptake in fried food products.

## CONFLICT OF INTEREST STATEMENT

The authors declare that they have no known competing financial interests or personal relationships that could have appeared to influence the work reported in this paper.

## Data Availability

The data that support the findings of this study are available from the corresponding author upon reasonable request.
